# Assays for Qualification and Quality Stratification of Clinical Biospecimens Used in Research: A Technical Report from the ISBER Biospecimen Science Working Group

**DOI:** 10.1089/bio.2016.0018

**Published:** 2016-10-01

**Authors:** Fay Betsou, Alexandre Bulla, Sang Yun Cho, Judith Clements, Rodrigo Chuaqui, Domenico Coppola, Yvonne De Souza, Annemieke De Wilde, William Grizzle, Fiorella Guadagni, Elaine Gunter, Stacey Heil, Verity Hodgkinson, Joseph Kessler, Michael Kiehntopf, Hee Sung Kim, Iren Koppandi, Katheryn Shea, Rajeev Singh, Marc Sobel, Stella Somiari, Demetri Spyropoulos, Mars Stone, Gunnel Tybring, Klara Valyi-Nagy, Gert Van den Eynden, Lalita Wadhwa

**Affiliations:** ^1^ Integrated BioBank of Luxemburg (IBBL), Luxembourg, Luxembourg.; ^2^ Biotheque-SML, Division of Genetics and Laboratory Medicine (DMGL), University Hospital of Geneva, Geneva, Switzerland.; ^3^ National Biobank of Korea, Cheongju, South Korea.; ^4^ Australian Prostate Cancer Bioresource/Queensland University of Technology, Brisbane, Australia.; ^5^ Cancer Diagnosis Program, Division of Cancer Treatment and Diagnosis (DCTD), National Cancer Institute, Rockville, Maryland.; ^6^ Moffitt Cancer Center, Department of Anatomic Pathology, University of South Florida, Tampa, Florida.; ^7^ University of California, San Francisco, AIDS Specimen Bank, San Francisco, California.; ^8^ Universitair Ziekenhuis Antwerpen, Edegem, Belgium.; ^9^ University of Alabama at Birmingham, Birmingham, Alabama.; ^10^ San Raffaele Rome University—IRCCS San Raffaele Pisana, Rome, Italy.; ^11^ Specimen Solutions, LLC, Tucker, Georgia.; ^12^ Coriell Institute for Medical Research, Camden, New Jersey.; ^13^ Cancer Research Division, Cancer Council NSW, Woolloomooloo, Australia.; ^14^ Medpace Reference Laboratories, Cincinnati, Ohio.; ^15^ Universitätsklinikum Jena, Jena, Germany.; ^16^ Department of Pathology, Chung-Ang University College of Medicine, Dongjak-gu, South Korea.; ^17^ Cellular Technology Ltd, Shaker Heights, Ohio.; ^18^ Precision for Medicine, Inc., Frederick, Maryland.; ^19^ Houston Methodist Research Institute, Biorepository, Houston, Texas.; ^20^ American Society for Investigative Pathology, Bethesda, Maryland.; ^21^ Biobank and Biospecimen Science Research, Windber Research Institute, Windber, Pennsylvania.; ^22^ Department of Pathology and Laboratory Medicine, Children's Research Institute, Medical University of South Carolina, Charleston, South Carolina.; ^23^ Blood Systems Research Institute, San Francisco, California.; ^24^ Karolinska Institutet, Stockholm, Sweden.; ^25^ University of Illinois Biorepository, Department of Pathology, College of Medicine, University of Illinois at Chicago, Chicago, Illinois.; ^26^ Department of Pathology and Cytology, GZA Hospitals, Wilrijk, Belgium.; ^27^ Baylor College of Medicine, Houston, Texas.

**Keywords:** quality control, biospecimen, qualification, tissue, cells, biological fluid

## Abstract

This technical report presents quality control (QC) assays that can be performed in order to qualify clinical biospecimens that have been biobanked for use in research. Some QC assays are specific to a disease area. Some QC assays are specific to a particular downstream analytical platform. When such a qualification is not possible, QC assays are presented that can be performed to stratify clinical biospecimens according to their biomolecular quality.

## Introduction

Clinical biospecimens used in research are subject to two types of laboratory analyses. The first of these is the analysis of established clinical biology/pathology parameters where reference ranges are usually known and methods are validated (e.g., CLIA or ISO15189 accreditation). Results of these analyses are necessary to support any research on novel clinically relevant biomarkers (definition of true positive and negative cases, use as a reference method). The second type is analysis of research parameters where there are usually no established reference ranges, and often methods are not validated by the laboratory as extensively as clinical biology/pathology methods.^1^ Results of these analyses are used to discover novel clinical endpoint correlates (biomarkers).

*In vivo* and *in vitro* pre-analytical variations have a more or less significant impact on the output of analyses, depending on the biospecimen type, the pre-analytical variable, and the analyte of interest. According to the type of analysis above, the word “significant” has a different meaning. In the first type—the analysis of clinical biology/pathology parameters—“significant” means clinically consequential at the diagnostic level. In the second type—analysis of research parameters—“significant” means statistically significant. Examples illustrating this concept are shown in Table 1.

**Table 1.** Examples Illustrating the Probable Impact of Pre-Analytical Conditions on the Analysis of Clinical or Research Parameters

*Pre-analytical condition*	*Biospecimen type*	*Analyzed parameter*	*Probable impact on the output of analyses*
Pre-centrifugation conditions	Serum	Clinical antibodies (e.g., anti-EBV IgG)	Non-significant (clinically)
Pre-centrifugation conditions	Serum	Research cytokines (e.g., IL-8)	Significant (statistically)
Pre-centrifugation conditions	Citrate plasma	Research cytokines (e.g., IL-8)	Non-significant (statistically)
Pre-centrifugation conditions	Citrate plasma	Coagulation parameters (e.g., factor V, factor VIII)	Significant (clinically)
Formalin fixation time	Lung tissue	IHC clinical antibodies (e.g., CK7)	Non-significant (clinically)
Formalin fixation time	Lung tissue	Mutation analysis by next-generation sequencing (e.g., allele frequency <10%)	Significant (not detectable mutation)
Alcohol fixation time	Lung tissue	Mutation analysis by next-generation sequencing (e.g., allele frequency <10%)	Non-significant (detectable mutation)

CK7, cytokeratin 7; EBV, Epstein–Barr virus; IgG, immunoglobulin G; IHC, immunohistochemistry; IL8, interleukin 8.

In some cases, the impact may be molecule- and even epitope-specific, for example tissue ischemia time may influence specific phospho-epitopes differently. A standard biospecimen research experimental protocol has been proposed for this type of research.^2^

Therefore, in all research comparing different groups of samples for biomarker discovery, it is critical that all samples are of comparable quality to avoid the introduction of uncontrolled variables and increase the power of analysis of biomarkers. There are two approaches to this end: either sample collections with careful pre-analytical annotations (SPREC),^3^ or retrospective collections with appropriate quality control (QC) and sample qualification or quality stratification. A combination of the two approaches to control compliance of procedures with specified SPRECs is also possible.

Biobanks underpin all three layers of biomarker discovery, validation, and use in clinical practice. In the biomarker discovery phase, biospecimens collected and processed with one Standard Operating Procedure (SOP), and corresponding to one quality category, should be used in order to avoid pre-analytical bias and increase the power of research. However, in the biomarker validation phase, biospecimens collected and processed with more than one known and documented SOPs and corresponding to more than one quality category should be used in order to validate the robustness of a biomarker to relevant pre-analytical variations. Finally, in the biomarker clinical implementation phase, biospecimens collected and processed via validated SOPs should be used in order to ensure successful and accurate clinical diagnostic results. For these reasons, during recent years, biobank managers, auditors, and funding bodies have been asking what assays can be performed in order to assess the quality of biospecimens objectively. This technical review provides answers to this question. Although gaps exist, this review shows that many tools are already available and can be used for specimen qualification.

## Methods

For the purposes of this technical report, the members of the International Society for Biological and Environmental Repositories (ISBER) Biospecimen Science Working Group held face-to-face meetings and teleconferences between 2013 and 2015. The chair of the Working Group performed a thorough literature review and compiled a list of relevant and effective QC attributes for different categories of biospecimens. This list was reviewed and complemented by members of the Working Group. When the information is based on published evidence, the corresponding reference is given. When no reference is given, the information corresponds to current practice or to the corresponding author's opinion.

The following definitions were used:

• *Biospecimen*: any biological specimen, which may be a:
  ○ *Primary sample*: specimen directly collected from the donor (e.g., whole blood, urine, solid tissue);
  ○ *Simple derivative*: sample prepared through a simple laboratory manipulation (e.g., after centrifugation of collection tubes or mechanical disruption of tissues) without the addition of chemical substances, and without cell disruption or cell selection as part of a multi-step process; or
  ○ *Complex derivative*: derivative whose isolation requires usage of multiple steps and/or addition of chemical substances (e.g., nucleic acids, proteins, lipids, sorted cells, cultured cells, immortalized cells).
• *Qualification*: process of examination of a biospecimen or a collection of biospecimens, and verification, based on objective analytical evidence, of their suitability for research use, either in a specific disease area or on a specific downstream analytical platform.
• *Quality stratification*: process of examination of a biospecimen or a collection of biospecimens, and their classification, based on objective analytical evidence, into distinct categories, each category corresponding to a specific *in vivo* biological characteristic (e.g., level of inflammation, % tumor, protein content) or to a specific *ex vivo* pre-analytical condition (e.g., pre-centrifugation conditions).
• *Biomolecular integrity*: quality status of a biospecimen, reflecting whether biomolecules of interest have not undergone either statistically or clinically significant changes relative to their *in vivo* state/levels.
• *Commutability*: equivalence of analytical methods, based on objective evidence.

The term “qualification” is used qualitatively. Therefore, a biospecimen is or is not qualified for use in research in a specific disease area or on a specific analytical platform.

The term “quality stratification” is used quantitatively. Therefore, one or more thresholds apply in order to stratify biospecimens in two or more quality categories. These quality categories correspond to defined *in vivo* or *in vitro* conditions.

**FIG. 1.**
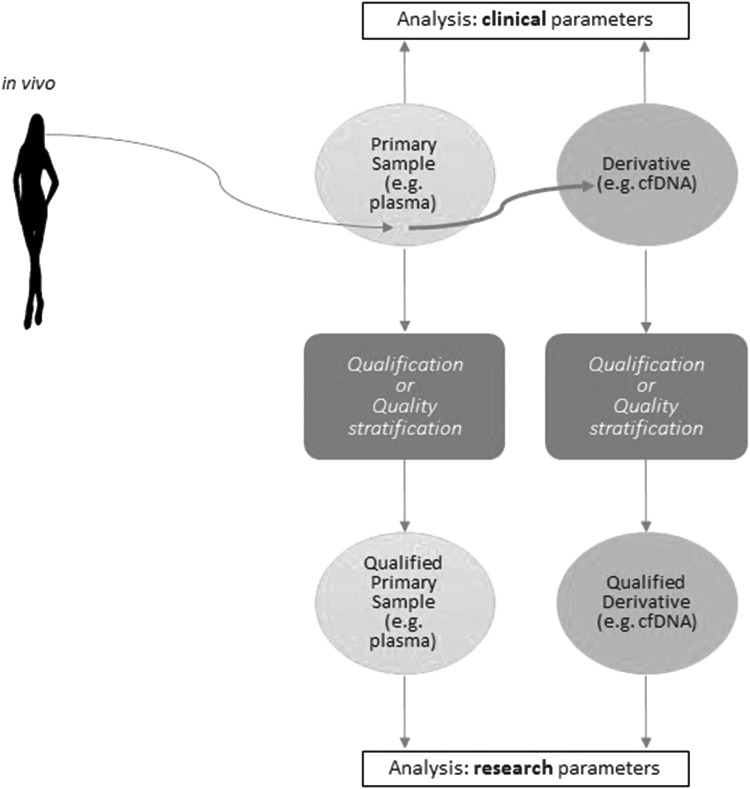
Flow diagram illustrating sample preparation and qualification for use in research.

When qualification is not possible because of lack of relevant assays, then quality stratification can be made. In some cases, qualification can be achieved for biomarker research in a specific disease area (Table 2) or on a specific downstream analytical platform. For primary samples, qualification depends on their biomolecular integrity. For simple or complex derivatives, qualification depends both on the biomolecular integrity of the primary sample from which the derivative has been extracted and on the efficiency/performance of the extraction, culture, cryopreservation, or other laboratory manipulation (e.g., cfDNA from plasma; Fig. 1).

**Table 2.** QC Measurands for Qualification for Use in Specific Disease Areas

*Biospecimen type*	*Measurand*	*Scope of qualification (disease area)*	*Measurement method*
Serum	Brain natriuretic peptide (BNP), NT-proBNP^6^ Angiopoietin-like 3 (ANFPTL3) Creatinine kinase MB isoenzyme (CK-MB) Endothelin 1 (ET-1)	Cardiovascular	EIA ECLIA/EIA EIA
Heparin plasma, serum	Matrix metalloproteinase-3 (MMP-3), matrix metalloproteinase-9 (MMP-9)		EIA
All plasma,^a^ serum	Troponin I & T		ECLIA/EIA
All plasma	Vasoactive intestinal peptide (VIP)		EIA
All plasma	Cholesterol ester transfer protein activity (CETP)	Lipid metabolism	Fluoroimmunoassay
Serum	Alanine aminotransferase (ALT)^7^	Liver	Enzymatic assay
Serum, all plasma	Tumor necrosis factor alpha (TNF-α)	Autoimmune, inflammatory	Sensitive EIA
Serum	Insulin C peptide^8^ Insulin-like growth factor II precursor	Endocrinology and diabetes	Fluoroimmunoassay, EIA/RIA
All plasma	Glucagon-like peptide 1 (cleared by DPP4)^9^		EIA/RIA
	Adenocorticotrophic hormone (ACTH)		ECLIA/RIA
All plasma, serum	Aldosterone Somatomedin C		EIA
Citrate plasma	Anti-factor Xa Fibrinogen	Coagulation	Clot detection
	Prothrombin fragments 1&2 Plasminogen activator inhibitor type 1 activity or antigen		EIA
	Thrombin generation assay		Fluoroimmunoassay
	Tissue-type plasminogen activator antigen (TPA antigen)		EIA
Urine	Beta 2 microglobulin	Nephrology	Nephelometry, EIA/RIA
All plasma, serum	Complement C3	Inflammation, immunology	Nepholometry, EIA
All plasma, serum	Intercellular adhesion molecule 1 (ICAM-1)		EIA
Citrate/heparin plasma, serum	TNF-α		EIA
Serum	M65 EpiDeath	Oncology	EIA
Heparin plasma, serum	Vascular adhesion molecule I (VCAM-1)		EIA
Serum	Mid-osteocalcin, osteocalcin, calcitonin	Musculoskeletal	ECLIA, EIA
	Parathyroid hormone, intact (PTH)		ECLIA, EIA
All plasma, serum	Telopeptide C terminal, type 1 collagen		ECLIA, EIA
Serum	Vitamin B12	Nutritional	ECLIA
CSF, serum, all plasma	Amyloid Ab42	Neurodegenerative	EIA
Serum, CSF	Neuron-specific enolase^10^		Kryptor immunoassay, EIA

^a^ All plasma refers to all EDTA, citrate, and heparinized plasma.

CSF, cerebrospinal fluid; DPP4, dipeptidylpeptidase 4; ECLIA, electrochemiluminescent immunoassay; EIA, enzyme immunoassay; QC, quality control; RIA, radioimmunoassay.

## Results

The results are presented in the form of Tables for fluid (Tables 3 and 4), tissue (Tables 5 and 6), and cytological biospecimens and their derivatives.

**Table 3.** QC Measurands for Qualification of Fluid Biospecimens and Their Derivatives

*Biospecimen type*	*Qualification parameter*	*Measurand*	*Scope of qualification*	*Measurement method*
Cf DNA	Contamination by blood cell DNA	DNA fragment size 100–300 bp^11^	Cf DNA genotyping	Microfluidic electrophoresis
Cf miRNA	Extraction efficiency	Spike in miRNA control (www.qiagen.com/lu/resources/resourcedetail?id=710c0168-e408-408b-95af-91df5b5b1dd6&lang=en)	Cf miRNA analysis	qRT PCR
		miRNA 16 or other ubiquitous miRNA target	Cf miRNA analysis	qRT PCR
Stool DNA	Inhibitors	SPUD^12^	PCR applications	qPCR
	Extraction efficiency	Bacterial DNA content	Bacterial DNA analysis	qPCR
		Human DNA content	Human DNA analysis	qPCR
Whole-blood cell DNA	Inhibitors	SPUD^12^	PCR applications	qPCR

Cf, cell free; qRT PCR, quantitative reverse transcription polymerase chain reaction.

**Table 4.** QC Measurands for Quality Stratification of Fluid Biospecimens and Their Derivatives

*Biospecimen type*	*Quality stratification parameter*	*Quality stratification parameter category*	*Measurand*	*Quality stratification threshold*	*Measurement method and reference*
Serum	Pre-centrifugation conditions	>8 h 4°C	Transferrin receptor	>300 IU/mL	ELISA^13^
	Post-centrifugation conditions	>24 h RT	sCD40L	<4 ng/mL	ELISA^14^
	Coagulation conditions	Not effectively coagulated	Fibrinogen	>100 mg/mL	ELISA
	Hemolysis	Hb contaminated	Hb	>50 mg/L	ELISA, spectrophotometry (www.ifcc.org/ifccfiles/docs/130401002end.pdf)
	Inflammation	Inflamed	C-reactive protein (CRP)	>10 mg/L	Nephelometry, ELISA
Rapid serum (RST)	Pre-centrifugation conditions	>48 h 4°C	Progastrin-releasing peptide (proGRP)	<30 pg/mL	Architect instrument^15^
EDTA plasma	Pre-centrifugation conditions	<3 h RT <2 h, 2–6 h, >6 h RT	Lacascore Metanomics	<5 MxP score ≥90, 89–70, <70	Enzymatic assays^16^ GC MS^17^
	Post-centrifugation conditions	>24 h RT	sCD40L	<0.3 ng/mL	ELISA (Betsou, unpublished)
All plasma^a^	Post-centrifugation conditions	>4 h RT	Complement component 3 peptide (C3f), complement component 4 (C4)	C4,1896.1*m*/*z*C3f, 2021.1*m*/*z*	MALDI-TOF-MS LC-ESI-MS/^18,19^
	Platelet contamination	Platelet poor	Platelets	<10^4^/mL	Cell count (https://en.wikipedia.org/wiki/Platelet-poor_plasma)
	Platelet activation	Activated platelets	β-thromboglobulin (βTG)	>200 ng/mL	ELISA^20^
	Hemolysis	Hb contaminated	Hb	>20 mg/L	ELISA, spectrophotometry^21^ (www.ifcc.org/ejifcc/vol13no4/13041002.htm)
	Inflammation	Inflamed	CRP	>10 mg/L	Nephelometry, ELISA
Citrate plasma	Pre-centrifugation conditions	>26 h 4°C	F VIII:C activity	<50 IU/dL	Coagulation activity assay^22^
	Post-centrifugation conditions	>9 years −80°C	Protein S activity	<50%	Coagulation activity assay^23^
Urine	Freezing	>6 months −20°C	Alkaline phosphatase activity	<0.1 IU/mmol creatinine	Enzymatic assay^24^
	Protein content	Low, intermediate, high, very high protein content	Creatinine Cystatin C	10, 50, 100 mg/dL 10, 50, 100 ng/mL	ELISA^25^
	acidity	Alcaline	pH	>8	pH paper
CSF	Post-centrifugation conditions	>32 h 4°C >3 months −20°C	Transthyretin (TTR) isoforms Cystatin C (CycC) truncation	Unmodified TTR-Cys10 peak <60% Intact CycC>truncated CysC peak	ESI-MS^26^ MALDI-TOF-MS, SELDI MS^27,28^
	Hemolysis	Hb contaminated	Hb	>15 ng/mL	ELISA^28^
Stool	Inflammation	Inflamed	Calprotectin	>50 mg/kg	ELISA^29^
Whole blood cell DNA	Double-strandedness	Highly double stranded	Spectrofluorimetry	>70%	Spectrophotometry, spectrofluorimetry
	Integrity	No degraded	MW	≥30 kb	Gel electrophoresis
		With no strand breaks	Long-range amplifiability	15 kb	PCR
	Purity	Not protein contaminated	A260/A280 ratio	≥1.5	Spectrophotometry
	Damage (oxidation, deamination, alkylation)	TBD	Apurinic/apyrimidinc sites	TBD	Colorimetric detection (aldehyde reactive probe-based)
	Post-bisulfitation quality	Of high DNA integrity	PCR amplicon size	≥600 bp	Multiplex PCR^30^
Whole blood cell RNA	rRNA integrity	Of high integrity	RIN	>7	Microfluidic electrophoresis
	mRNA integrity	Not 5′ degraded	mRNA index	|ΔCt|<1	qRT PCR^31^
	purity	Not protein contaminated	A260/A280 ratio	>1.6	Spectrophotometry
	Pre-centrifugation conditions	>24 h RT	Gene targets^b^	TBD	qRT PCR^32,33^
	WBC subpopulation composition	Normal composition	Lymphocytes, granulocyte, monocyte numbers	Neutrophils: 2.5–7.5 × 10^9^/L Lymphocytes: 1.5–3.5 × 10^9^/L Monocytes: 0.2–0.8 × 10^9^/L	Blood count^34^ (http://emedicine.medscape.com/article/2085133-overview)

^a^ All plasma refers to all EDTA, citrate, and heparinized plasma.

^b^ Under investigation by the International Society for Biological and Environmental Repositories (ISBER) Biospecimen Science Working Group.

ELISA, enzyme-linked immunosorbent assay; Hb, hemoglobin; LC-ESI-MS, liquid chromatography electrospray ionization mass spectrometry; MALDI-TOF-MS, matrix-assisted laser desporption/ionization time of flight mass spectrometry; RT, room temperature; SELDI MS, surface-enhanced laser desorption/ionization mass spectrometry; TBD, to be defined; WBC, white blood cell.

**Table 5.** QC Measurands for Qualification of Tissue Biospecimens and Their Derivatives

*Biospecimen type*	*Qualification parameter*	*Measurand*	*Scope of qualification*	*Measurement method*
Frozen tissue	Freeze–thaw	Cell lysis	IHC, RNA-based analyses	H&E staining
Viable frozen tissue	Sterility	Absence of contaminants	Tissue culture	Growth on agar; mycoplasma testing
	Cryopreservation conditions	Post-thaw viability		Growth in flasks

H&E, hematoxylin and eosin.

**Table 6.** QC Measurands for Quality Stratification of Tissue Biospecimens and Their Derivatives

*Biospecimen type*	*Quality stratification parameter*	*Quality stratification parameter category*	*Measurand*	*Quality stratification threshold*	*Measurement method and reference*
Tumor	% tumor	Tumor-rich	Tumor	>70%	H&E staining, digital pathology
FFPE	Fixation time NBF	>72 h	None to date^a^	TBD	qRT PCR
	Fixation conditions	NBF (no acidic formalin)	Size range RT PCR	∼250 bp	RT PCR
	Cold ischemia	>12 h	None to date^a^	TBD	qRT PCR
Frozen tissue	Cold ischemia	>12 h	None to date^a^	TBD	qRT PCR
FFPE DNA	Fixation conditions (cross-linking); extraction efficiency DNA integrity	Highly deaminated	qPCR ΔCt	ΔCt ≥1.55	Illumina FFPE QC kit Agilent NGS FFPE QC kit or equivalent^42^
		CGH compatible WGA compatible	PCR amplicon size	≥200bp, ≥300 bp	Multiplex PCR^43,44^
		Of good integrity	WGA score	≥3 μg yield	WGA (www.enzolifesciences.com/ENZ-42440/bioscore-screening-and-amplification-kit-20-reactions)
		Of good integrity	DIN	>7	Microfluidic electrophoresis
FFPE RNA	mRNA integrity	Extremely 5′ degraded	mRNA index	|ΔCt|>8	qRT PCR^31^
		Of good mRNA integrity	Size range RT PCR	∼250 bp	RT PCR
	Fixation time	>72 h	Gene targets^a^	TBD	qRT PCR
	Ischemia time	>12 h	Gene targets^a^	TBD	qRT PCR
FFPE proteins	Ischemia time	TBD	Phospho-Tyrosine (P Tyr 100)	TBD	IHC^45^
Frozen tissue DNA	Processing/storage conditions; extraction efficiency	With no strand breaks	Long range PCR	15 kb	PCR
Frozen tissue RNA	Processing/storage conditions; extraction efficiency rRNA integrity	Of high integrity	RIN RIS DV200 or equivalent	>6	Microfluidic electrophoresis (www.agilent.com/cs/library/applications/5989-1165EN.pdf), (www.qiagen.com/gb/shop/automated-solutions/dna-analysis/qiaxcel-advanced-system/), (www.aati-us.com/product/fragment-analyzer/download_dv200_metric)
	mRNA integrity	Not 5′ degraded	mRNA index	|ΔCt|<1	qRT PCR^31^
	Purity	Not protein contaminated	A260/A280 ratio	>1.6	Spectrophotometry
Frozen tissue proteins	Postmortem interval/ischemia	>48 h cold ischemia	αII spectrin cleavage (no 285 kDa, only 150 kDa)	285 kDa >150 kDa	Western blot^46^

^a^ Under investigation by the ISBER Biospecimen Science Working Group.

FFPE, formalin-fixed, paraffin-embedded; NBF, normal buffered formalin.

Table 2 includes information on QC measurands for qualification for use of samples in specific disease areas.^4,5^ The measurands in this table are molecules that are recognized biomarkers in the respective disease areas and are also known to be labile. Detection of the measurand above the method's level of detection is necessary (though not always sufficient) for qualification of a sample. As an example for reading Table 2, if Aβ42 is undetectable in CSF samples, then these samples cannot be qualified for research in the area of neurodegenerative diseases.

Tables 3, 5, and 7 include information that can be used for the qualification of fluid, tissue, or cytological specimens, respectively, in the scope of different types of downstream analyses. In these tables, “qualification parameter” is the quality aspect of the biospecimen that is being evaluated; “measurand” is the molecule, or the morphological or functional characteristic that is being measured and whose positive or negative result is necessary for the qualification; “scope of qualification” is the type of downstream analysis for which the biospecimen is being qualified as fit-for-purpose; and “measurement method” is the type of method that is used to measure the measurand.

**Table 7.** QC Measurands for Qualification of Cytological Biospecimens

*Biospecimen type*	*Qualification parameter*	*Measurand*	*Scope of qualification*	*Measurement method*
All cell suspensions	Sterility	Absence of contaminants	Culture	Growth on agar; mycoplasma testing
	Identity	Protein markers Genetic identity	Any type of downstream analysis	ICC, ELISA, FC PCR, STR genotyping, FISH, karyology
	Purity	Absence of protein markers Absence of cellular impurities	Any type of downstream analysis	ICC, ELISA, FC FC
	Genomic stability	Chromosomal stability Phenotypic stability	Any type of downstream analysis	G-banding, ICC, FC, microscopy
Cell line	Identity	STR, karyotype, SNP fingerprint^47^	Any type of downstream analysis	PCR, karyology/FISH, sequencing/arrays
Stem cells	Sterility	Absence of contaminants	Culture, functional assays	Growth on agar; mycoplasma testing, HIV, HBV, HCV, EBV, CMV, syphilis, fungus, bacteria, endotoxin
	Normal karyotype	Karyotype	Any type of downstream analysis	G-banding
	Identity matching	Match parent cells	Any type of downstream analysis	STR
	Non oncogenicity	C-Myc, P53, p21, p16 absence of expression	Any type of downstream analysis	Immunostaining, gene expression
Lymphoblastoid cell lines (LCL)	Normal karyotype	Karyotype	Any type of downstream analysis	G-banding
	EBV transformation	EBV gene expression	Any type of downstream analysis	RT PCR^48^
Circulating tumor cells (CTC)	Cancer phenotype	EpCam+, CK8+, 18+, 19+, CD45–	Any type of downstream analysis	Immunostaining^49^

CMV, cytomegalovirus; FC, flow cytometry; FISH, fluorescent in situ hybridization; HBV, hepatitis B virus; HCV, hepatitis C virus; HIV, human immunodeficiency virus; ICC, immunocytochemistry; SNP, single nucleotide polymorphism; STR, short tandem repeats.

Tables 4, 6, and 8 include information that can be used for the quality stratification of a fluid, tissue, or cytological biospecimen, respectively. In these tables, “qualification parameter” is the quality aspect of the biospecimen for which the biospecimen is being stratified; “measurand” is the molecule, or the morphological or functional characteristic that is being measured and whose level is used to stratify the biospecimens in categories; “quality stratification thresholds” are the levels of the measurand, which are critical for the quality stratification; and “measurement method” is the type of method that is used to measure the measurand. The quality stratification thresholds listed in Tables 4, 6, and 8 classify the biospecimens into the categories of the qualification parameter given. The “time xxx/temperature yyy” categories correspond to available experimental data, but they should be understood as “time xxx/temperature yyy or equivalent conditions.” The quality stratification thresholds listed in Tables 4, 6, and 8 are those corresponding to the measurement methods described in the references. Application of a threshold with a measurement method that is different from the method that has been used for the establishment of the threshold requires previous demonstration of the commutability of the methods.

**Table 8.** QC Measurands for Quality Stratification of Cytological Biospecimens

*Biospecimen type*	*Quality stratification parameter*	*Quality stratification parameter category*	*Measurand*	*Quality stratification threshold*	*Measurement method and reference*
Peripheral blood mononuclear cells (PBMCs)	Cryopreservation	Of high viability	Post thaw viability	>80%	FC; trypan blue
	Specificity (granulocyte contamination)	<12–14 h RT post venipuncture; With no T-cell function inhibition	CD15+ granulocytes	<20%	FC^50^
All cell suspensions	Biological activity	Cell type specific	Receptors Secreted proteins mRNA expression Migration	Cell type-specific	ICC, FC, microscopy, FRET microscopy, ELISA, qRT PCR, microarray Dunn, Boyden or Impedance Chamber, Scratch assay, Matrigel invasion assay
	Concentration, viability	Of high viability	Cell number Viability	>80%	FC, impedance, microscopy Viability assays
Sperm	DNA integrity	Of compromised DNA integrity	Acridine Orange staining and acid-induced denaturation	COMPa_t_^a^ >30%	Sperm chromatin structure assay^51^
Viable RBC	Storage lesion	>4 days 4°C	2,3-diphosphoglycerate (2,3-DPG)	<2 mmol/L	Spectrophotometry (340 nm)^52^
Viable platelets	Activation	With highly activated platelets	Surface P selectin (CD62)	>70%	Flow cytometry^53^
Stem cells	Cryopreservation conditions	Efficiently cryopreserved	Colony formation and diameter doubling	<5 days	Colony doubling
	Surface antigen expression of stem cell markers	Stem cell positive	expression SSEA-4, expression SSEA-1	>80%, <20%	Immunostaining
	Pluripotency	Pluripotent	Upregulation of genes associated with each of the three germ layers	2-fold compared to control (at least one gene per germ layer)	qRT PCR
Liquid biopsy-based cytology specimens	Cell concentration	*Downstream application-specific*	Number of cells	*Downstream application-specific*	Cell count
Sorted cells	Purity	Pure	% of cells with expected immunophenotype, e.g., T cells (CD3), NK cells (CD16/56), B cells (CD19/20), monocytes (CD14), functional memory B cells (CD19, CD27, CD45, CD38, CD138)	>90%	Flow cytometry

^a^ COMP, cells outside the main population.

FRET, fluorescence resonance energy transfer; RBC, red blood cell; SSEA, stage-specific embryonic antigen.

### Tissue type specificities

Assays for tissue qualification or quality stratification may be tissue type–specific. Some examples are given below. Fixation conditions have a significant impact on P-Akt and P-Erk1/2 in breast cancer tissue.^35^ Ischemia has a significant impact on estrogen and progesterone receptors in breast tissue.^36,37^ A Tissue Quality Index has been proposed for formalin-fixed, paraffin-embedded breast tissue in order to assess its cold ischemia time by immunohistochemistry.^38^ Stathmin^2–20^ has been proposed as indicator of degradation in brain tissue by matrix-assisted laser desorption/ionization time of flight mass spectrometry.^39^ AKT-P has been proposed as indicator of postmortem conditions in brain tissue by western blot.^40^ Superoxide dismutase in the liver and peptidyl-prolyl-cis-trans isomerase and insulin C-peptides in the pancreas have been associated with postmortem delay and assessed by two-dimensional difference in gel electrophoresis.^41^

## Discussion

**FIG. 2.**
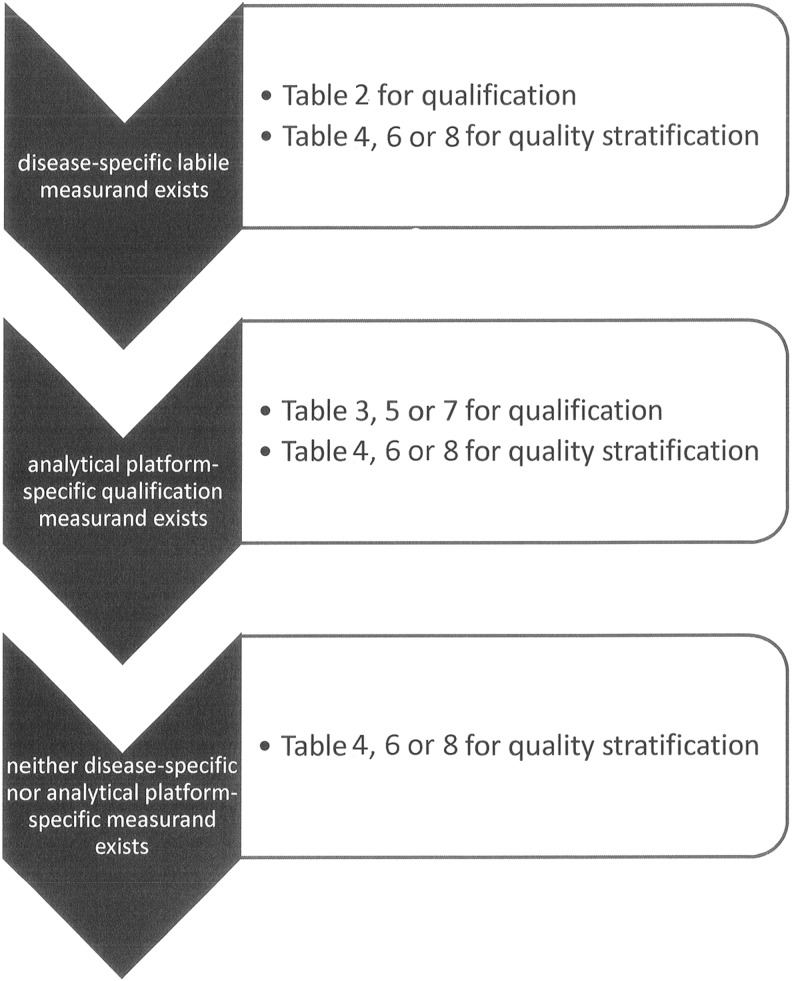
Decision tree for any given specimen type.

This article proposes a biospecimen QC strategy, based on current state of knowledge, in the form of summary tables (Fig. 2).

The qualification and quality stratification assays presented in this technical report do not aim for an absolute assessment of the quality of samples, since a sample can be of high enough quality (fit-for-purpose) for one type of analysis (e.g., antibody analysis), but not for other types of analyses (e.g., metabolite analysis). Therefore, scientists should devote time and effort to understand and define what sample quality is needed to obtain consistent results with a given downstream analytical platform. As can be seen from Tables 3, 5, and 7, there are several gaps in the area of biospecimen qualification for use on specific analytical platforms. These include, for example, urine, saliva, or frozen tissue qualification for use in proteomic analyses, serum, plasma, or other body fluid qualification for use in miRNome analyses, or DNA qualification for use in methylation analyses. In the absence of such knowledge, this technical report offers a strategy for sample quality stratification so that bias due to samples of inconsistent quality levels can be minimized.

The information provided in this report is important because its application will enable and support bioprocessing method validation by providing relevant readouts (measurands); assessment of the quality of biospecimens of unknown history; biomarker discovery by ensuring use of qualified biospecimens or biospecimens belonging to a specific quality category; validation of biomarker robustness by using quality-stratified biospecimens belonging to different, defined quality categories; implementation of novel biomarkers in clinical practice; and characterization and production of clinical reference materials.

For the above purposes, QC measurands of clinical biospecimens can be assessed either by the biobanks themselves, or by subcontractors/collaborators who are accredited or successfully participate in relevant Proficiency Testing schemes. The results of the QC can be used by biobanks for qualification of legacy collections (the definition of cutoff values for acceptance of legacy collections or specific samples can be made and disclosed by the biobank), by end users for stratification of samples of different origins, or by funding agencies for assessment of the fitness for purpose of collections to be used in the context of grant allocation.
